# The Relationship Between Digital Game Addiction, Computational Thinking Skills, and Well-Being of Middle School Children With and Without Congenital Heart Disease

**DOI:** 10.3390/children13050686

**Published:** 2026-05-16

**Authors:** Emine Akkaş Baysal, Nezahat Hamiden Karaca, Ayhan Pektaş

**Affiliations:** 1 Child Development Department, Sandıklı School of Applied Sciences, Afyon Kocatepe University, 03204 Afyonkarahisar, Türkiye; 2 Faculty of Education, Early Childhood Education, Afyon Kocatepe University, 03204 Afyonkarahisar, Türkiye; 3 Department of Pediatric Cardiology, Afyonkarahisar Health Sciences University Hospital, 03200 Afyonkarahisar, Türkiye; ayhan.pektas@afsu.edu.tr

**Keywords:** congenital heart disease, digital game addiction, computational thinking, well-being

## Abstract

**Highlights:**

**What are the main findings?**

- This study aims to examine associations within and across middle school children with and without congenital heart disease in terms of digital game addiction, computational thinking skills, and well-being.
- Children with congenital heart disease show different patterns in well-being and digital gaming behaviours compared to healthy peers.

**What is the implication of the main finding?**

- The findings suggest that children with congenital heart disease may require tailored educational and digital well-being support programs to promote healthy technology use.
- Schools and healthcare professionals should consider integrating computational thinking development with digital well-being interventions for children.

**Abstract:**

**Background:** Digital gaming is an integral part of children’s everyday lives and may relate to both cognitive development and psychosocial well-being. Although computational thinking is considered a key skill for navigating digital environments, limited research has examined how digital game addiction relates to computational thinking and well-being, particularly among children with chronic health conditions such as structural heart disease. **Objectives:** This study aimed to examine the associations among digital game addiction, computational thinking skills, and well-being among middle school children with structural heart disease and their typically developing peers. **Methods:** An exploratory correlational design was employed. The sample consisted of 30 children with structural heart disease and 25 typically developing peers aged 10–14 years. Data were collected using the Computational Thinking Skills Scale, the Digital Game Addiction Scale for Children, and the EPOCH Well-Being Scale. Relationships among variables were examined using correlation analyses. **Results and Conclusions**: Among children with structural heart disease, higher levels of digital game addiction were associated with lower creativity, collaboration, and overall computational thinking skills. Computational thinking skills were positively associated with selected dimensions of well-being, particularly connectedness and perseverance. In typically developing children, digital game addiction was negatively associated with several computational thinking dimensions, whereas perseverance and certain aspects of well-being were positively associated with computational thinking skills. Overall, digital game addiction showed limited associations with well-being in both groups. These findings suggest that the relationships among digital game addiction, computational thinking, and well-being may be complex and context-dependent. Given the exploratory correlational design and relatively small sample size, the results should be considered preliminary.

## 1. Introduction

A significant portion of chronic diseases observed in childhood consists of congenital heart diseases. Congenital heart diseases may affect children’s daily activities, participation in physical play, and social relationships, increasing the importance of psychosocial well-being and quality of life in pediatric cardiology. Children with structural heart disease often undergo long-term medical follow-up and, in some cases, invasive procedures such as angiography or cardiac surgery. These experiences may create physical, emotional, and social challenges that affect children’s development. Due to health-related restrictions, many children with structural heart disease may spend more time engaging in sedentary and screen-based activities compared to their peers. In particular, digital games may become a prominent source of entertainment, socialisation, and daily engagement for these children. Although digital games may provide cognitive and recreational benefits, excessive and uncontrolled use may also increase the risk of problematic gaming behaviours.

Digital games can support the development of skills such as problem-solving, strategic thinking, and hand–eye coordination. However, excessive digital game use has also been associated with behavioural addiction and may negatively relate to children’s social and emotional well-being. Although excessive or problematic gaming has been associated with negative outcomes, recent empirical studies have also suggested that moderate and structured digital game use may support certain cognitive and motivational processes in children, including problem-solving skills, attention regulation, and engagement in learning-related tasks [1,2].

At the same time, children’s engagement with digital environments has also increased interest in computational thinking, one of the core competencies associated with 21st-century learning [3]. Considered as essential as reading and writing, computational thinking underpins coding and programming education [4,5]. Due to the growing importance of digital literacy and problem-solving skills, many educational systems have increasingly integrated computational thinking into curricula from early ages [6]. Based on these considerations, the present study aimed to explore the associations among digital game addiction, computational thinking skills, and well-being in middle school children with structural heart disease and their typically developing peers.

## 2. Literature Review

### 2.1. Congenital Heart Disease and Child Development

Congenital heart diseases (CHD) are defined as anomalies that occur due to the incomplete or irregular structural and functional development of the cardiovascular system during the embryonic period. According to the literature, approximately 8–12 per 1000 live births are affected by some form of congenital heart disease, making CHD one of the most common congenital anomalies in childhood [7,8]. If untreated in the early period, CHDs carry significant risks of morbidity and mortality, and about half of affected neonates receive a diagnosis within the first week of life [9,10]. According to the Turkish Statistical Institute [11], approximately 12,000–13,000 infants are born with CHD each year, and circulatory system diseases rank among the leading causes of mortality in the country. Similarly, the American Heart Association [12] reports that the prevalence of CHD ranges between 2.4 and 13.7 per 1000 live births. These figures indicate that congenital cardiac anomalies represent a critical public health issue for childhood health.

Clinical manifestations of CHD vary according to the child’s age. For instance, infants often exhibit poor weight gain, inadequate nutrition, and cold sweating, whereas older children may experience fatigue, respiratory distress, and periorbital oedema [13]. Children with CHD must cope with their condition for many years, which negatively affects their growth and developmental periods. So, their quality of life decreases, daily activities are restricted, and social interactions with peers may be impaired [14,15].

Advances in medical and surgical care have increased the survival rates of children with CHD; however, due to the complex nature of heart disease, cognitive, nutritional, and developmental challenges persist [16]. Particularly following early surgical interventions, delays in motor development, growth problems, and increased risk of infections are frequently observed. For example, Cooper et al. [17] reported that infants who underwent CHD surgery had lower height and weight values at four months compared to healthy peers, while another study on neurodevelopmental outcomes in infants with congenital heart disease has shown that motor development in these infants tends to lag behind that of healthy infants [18]. Moreover, Uzark et al. [19] reported that children with heart disease had significantly lower quality of life scores in both physical and psychosocial domains, and even among children with mild cardiac conditions, 19.2% exhibited significant impairment in psychosocial functioning.

Recent studies have further indicated that children with congenital heart disease may be at increased risk for subtle neurodevelopmental and psychosocial difficulties, including challenges in cognitive functioning, social cognition, and emotional adjustment. These outcomes are increasingly understood within a neurodevelopmental framework that considers both medical and environmental influences on developmental trajectories [20,21]. These findings indicate that children with CHD are at risk not only physiologically but also in terms of cognitive and psychosocial development. Moreover, the child’s developmental stage determines the extent of these impacts, with some age groups demonstrating more pronounced growth and developmental challenges [16]. Therefore, addressing the effects of CHD on child development necessitates a biopsychosocial approach, which is of critical importance for both healthcare professionals and researchers.

### 2.2. Digital Game Addiction

The rapid advancement of technology has created a world in which individuals can meet their needs more quickly and conveniently. While this transformation offers many advantages, excessive or uncontrolled use of technology may also be related to adverse outcomes. In particular, problematic use of technological devices has given rise to a relatively new phenomenon within behavioural addictions, commonly referred to as “technological addiction” [22]. Within this framework, technological addiction can be increasingly considered a significant risk factor in children’s developmental processes.

Digital games can provide diverse opportunities for children’s cognitive and social development. Studies have demonstrated that such games may enhance literacy skills, improve hand–eye coordination and visuospatial abilities, support creativity, and strengthen problem-solving skills [23]. Furthermore, digital games have been associated with stress reduction, increased motivation, enjoyable leisure activities, and opportunities for online social interaction [24]. These findings indicate that, when used appropriately and within limits, digital games may serve as supportive tools in child development. However, digital game addiction has increasingly become a critical concern, particularly among children and adolescents. It is defined as a behavioural pattern characterised by excessive and uncontrolled engagement [25,26]. At this point, digital game addiction appears to overshadow the potential benefits of technology use by posing significant developmental risks.

The consequences of addiction manifest in physical, psychological, and social domains. Prolonged sedentary behaviour in front of screens may be related to musculoskeletal problems, circulatory disorders, obesity, vision impairments, and sleep disturbances [27]. Among adolescents, excessive gaming may also be linked to reduced face-to-face communication, weakened social relationships, feelings of loneliness, and diminished sensitivity toward social issues [28]. Thus, digital game addiction may be related not only to individual well-being but also to broader patterns of social functioning.

Empirical evidence further highlights the relationship between game addiction and problematic behaviours. Bağatarhan [29] reported that higher tendencies toward digital game addiction are associated with increased externalising and internalising behaviour problems, antisocial tendencies, and egocentric behaviour. Similarly, Manap and Durmuş [30] emphasised that healthy family roles and digital parenting awareness are associated with more positive outcomes. In contrast, negative parental modelling and digital neglect exacerbate the risk of addiction. These findings underscore that digital game addiction is influenced not only by individual tendencies but also by family dynamics and parental practices.

Recent empirical studies have increasingly emphasised the role of emotional regulation processes in the development and maintenance of problematic gaming behaviours in children and adolescents. Difficulties in emotional regulation have been associated with higher levels of problematic gaming and maladaptive coping through digital game use, particularly in pediatric and adolescent populations [31,32]. These findings suggest that gaming behaviours may be closely linked to affective self-regulation mechanisms rather than being solely behavioural or recreational phenomena. In conclusion, digital games, when used in moderation, may positively contribute to children’s cognitive, social, and emotional development. However, when overused, they can evolve into an addictive behaviour that undermines children’s health, academic performance, and social adjustment. Therefore, digital game addiction should be regarded as a critical issue that requires attention at both individual and societal levels.

### 2.3. Computational Thinking Skills

Computational thinking (CT) is regarded as an important 21st-century competency encompassing problem-solving, logical reasoning, abstraction, and algorithmic thinking [5]. Subsequent work reconceptualised CT for educational contexts, arguing that CT is not only for computer scientists but is a transferable cognitive toolkit for K–12 learners [4,33]. More recent research has also highlighted the relevance of computational thinking within broader cognitive and developmental frameworks, particularly in relation to executive functions and problem-solving abilities in digital environments. Emerging evidence suggests that computational thinking skills may be associated with cognitive flexibility, planning, and self-regulation, which are critical for adaptive functioning in children and adolescents [34].

In applied and curricular research, CT is commonly operationalised as a set of component skills. Several frameworks identify overlapping elements such as (a) problem definition and decomposition, (b) data collection/representation and pattern recognition, (c) algorithmic planning and sequencing, (d) implementation and debugging, and (e) evaluation/refinement [35,36]. Kalelioğlu et al.’s [35] five-stage model explicitly maps classical problem-solving phases to CT components—a useful conceptual bridge for linking CT to other cognitive constructs, such as computational reasoning and mathematical problem solving. Thus, conceptualising CT through these component processes not only clarifies its structure but also provides a pedagogical pathway for integrating computational reasoning into broader domains of cognitive and academic development.

Evidence from reviews and empirical studies shows both promise and important caveats for CT instruction. Systematic and narrative reviews report that CT activities can strengthen problem-solving, sequencing, and debugging skills—especially when pedagogy is developmentally appropriate and scaffolded—but effects vary by age, program design, and teacher preparedness [3,37,38]. Early childhood and elementary interventions often emphasise play-based, narrative programming (e.g., ScratchJr, KIBO) to foster foundational CT concepts without imposing advanced syntactic programming demands [3,38]. This evidence underscores that the success of CT education depends less on the specific tools used and more on how effectively instruction aligns with learners’ cognitive readiness, motivation, and real-world problem contexts.

Policy-oriented assessments reinforce the importance of CT-related competencies for broader educational goals. The OECD’s PISA 2015 assessment introduced collaborative problem-solving and problem-solving skills, prompting calls for curricular reforms that integrate digital problem-solving from early grades [6,39]. This policy backdrop helps explain why many countries and high-impact studies (including those published in Q1/Q2 journals) prioritise teacher professional development and systemic supports to embed CT across curricula [4,39]. Consequently, computational thinking is increasingly viewed not merely as a technical skill but as a foundational literacy—essential for preparing students to navigate, adapt, and innovate in digitally mediated learning environments.

For research linking CT to psychosocial outcomes—such as well-being or behavioural adaptation—the literature is thinner but growing. Several studies suggest that CT activities can promote collaborative problem solving and resilience in learning contexts. At the same time, other lines of research highlight substitution effects (e.g., excessive screen time) that may disrupt socio-emotional development if technology is used without pedagogical mediation [40,41]. This mixed evidence signals that CT’s developmental benefits depend heavily on context: the learning environment, the balance between guided vs unguided digital play, and teachers’ capacity to integrate CT into meaningful, socially interactive tasks.

### 2.4. Well-Being Level

Well-being in children is a multidimensional construct encompassing psychological resilience, subjective happiness, and social-emotional development [42,43]. Psychological resilience refers to the capacity to adapt positively in the face of adversity, stress, or risk, and is considered a positive outcome in the context of psychological difficulties [44]. Studies have shown that higher resilience in children correlates with fewer internalising and externalising problems [44].

Subjective happiness or life satisfaction in children is another core component of well-being. The broaden-and-build theory of positive emotions posits that positive affect broadens cognitive and behavioural repertoires, which, over time, builds enduring psychological resources. In educational settings, happier children tend to show greater engagement and social competence and lower stress [45]. Social-emotional development includes emotional regulation, social skills, empathy, and interpersonal competence. Emotion regulation is a central mechanism linking resilience and social-emotional adjustment: children who can better regulate their emotions are more likely to exhibit social well-being and experience fewer behavioural problems [46]. For instance, in preschool children, social and emotional well-being and resilience predicted behavioural issues, with partial mediation by emotion regulation [46]. Overall, nurturing children’s positive emotions and their capacity for emotional regulation can strengthen their adaptive functioning, helping them build the psychological and social foundations necessary for lifelong well-being.

Interventions based on Social and Emotional Learning (SEL) have demonstrated positive effects on children’s well-being: they enhance emotional regulation and social competence and reduce maladaptive behaviours [47]. These improvements in social-emotional capacity feed back into resilience and subjective well-being, forming a positive cycle.

### 2.5. Relationships Between Variables: Digital Game Addiction, Computational Thinking and Well-Being

In today’s technology-saturated world, digital games have become one of the primary environments through which children learn, communicate, and express themselves. While moderate game-based engagement can foster creativity and problem-solving skills, excessive or uncontrolled use has raised serious concerns regarding problematic gaming behaviours and psychosocial risks in early and middle childhood [48,49]. Although prior studies have examined digital gaming behaviours, computational thinking, and child well-being separately, limited research has examined their associations within the same sample. In the present study, computational thinking is conceptualised as a cognitive skill domain that may co-vary with digital gaming tendencies and indicators of well-being. Accordingly, this study was designed as an exploratory correlational investigation of associations among these variables.

Recent studies emphasise the growing interconnection among children’s digital behaviours, cognitive problem-solving abilities, and psychological well-being. As digital gaming becomes an integral part of children’s daily lives, it may serve both as a learning environment and a potential risk context, depending on its intensity and purpose of use [48,49]. Therefore, understanding how digital game addiction relates to computational thinking and well-being may provide valuable insights into children’s adaptation in the digital age.

Digital game addiction has been associated in the literature with various negative psychological outcomes, including increased depression, anxiety, loneliness, and decreased life satisfaction [50,51]. Excessive gaming may also interfere with social relationships, sleep quality, and academic engagement, which are important components of emotional and social well-being [52]. From a psychological perspective, compulsive gaming behaviours are often linked to reward-based mechanisms that may reduce intrinsic motivation and emotional regulation capacities [53]. Accordingly, higher levels of gaming addiction are generally associated with lower indicators of psychological well-being, including resilience and subjective happiness [44].

In contrast, computational thinking—a structured cognitive process involving problem decomposition, algorithmic reasoning, and logical abstraction—has been associated with positive cognitive and behavioural outcomes, such as self-efficacy, persistence, and metacognitive awareness [37,54]. When children engage in digital activities with purposeful learning intentions (e.g., coding, design, or simulation), they may develop adaptive digital engagement rather than compulsive patterns [55]. CT can thus serve as a positive outcome in the context of maladaptive digital behaviours by enhancing executive functions, including inhibitory control and goal-directed behaviour [56]. These cognitive mechanisms support emotional regulation and coping strategies, which are key components of well-being.

Emerging evidence suggests a bidirectional or mediating relationship among these variables. While excessive gaming impairs cognitive control and well-being, well-developed computational thinking skills may exacerbate this negative impact by promoting the reflective use of technology [56,57]. In other words, children with higher CT levels are more likely to use games strategically for exploration, problem solving, and creativity rather than for escapism or emotional compensation [58]. Previous studies have shown that problematic gaming behaviours may be associated with distinct motivational profiles, particularly escapism-oriented gaming motivations. In this context, escapism has been described as a coping-oriented motivation in which digital gaming is used to avoid stress, negative emotions, or real-life difficulties, and higher escapism motives have been associated with increased risk of problematic gaming behaviours. In addition, the literature suggests that parental mediation strategies may differentially influence children’s gaming-related outcomes. Restrictive mediation strategies may reduce gaming time in the short term. However, they may not consistently prevent problematic gaming behaviours. In contrast, active mediation and co-use approaches have been associated with healthier gaming experiences, improved communication, and more adaptive self-regulation among children and adolescents. From a theoretical perspective, this relationship aligns with Bandura’s social cognitive theory, which emphasises the role of self-regulation and mastery experiences, and with Self-Determination Theory, which posits that autonomy and competence contribute to both learning motivation and psychological well-being [59]. Although prior literature suggests that computational thinking may mediate or moderate the relationship between digital game addiction and well-being, the present study does not test these models. Instead, it adopts an exploratory correlational approach to examine associations among these variables without assuming directionality or causality. Based on prior literature, statistical associations among digital game addiction, computational thinking, and well-being were expected; however, no directional or causal assumptions were tested. Specifically:

- DGA is expected to be negatively associated with well-being through its relationship with emotional and social functioning,
- CT is expected to be positively associated with well-being through cognitive control and self-regulation processes,
- CT and DGA are expected to show an inverse association due to differences in self-regulatory capacity.

From a theoretical perspective, the relationship between digital game addiction, computational thinking, and well-being can be better understood through cognitive-developmental and self-regulatory frameworks. Computational thinking is not only a set of problem-solving skills but also reflects higher-order cognitive processes closely related to executive functions, such as planning, cognitive flexibility, and inhibitory control. These processes are also central to self-regulation, which has been consistently associated with both adaptive digital media use and psychological well-being in children and adolescents. Within this framework, excessive or maladaptive engagement in digital gaming may be linked to differences in self-regulatory capacities, which in turn may be reflected in computational thinking performance and broader psychosocial outcomes. Accordingly, computational thinking is conceptualised in this study as a potential cognitive marker that may co-vary with both digital gaming behaviours and well-being indicators within a shared developmental context.

Although previous studies have separately examined digital gaming behaviours, computational thinking, and well-being, limited research has examined their associations within the same population, particularly among children with structural heart disease. Moreover, existing studies have largely focused on general adolescent populations, while children with chronic health conditions remain understudied. Therefore, the present study aimed to explore the associations among digital game addiction, computational thinking skills, and well-being in middle school children with structural heart disease and their typically developing peers. The research questions of the study are as follows:

1. What associations exist between digital game addiction and computational thinking skills among middle school children with structural heart disease and their typically developing peers?
2. What associations exist between digital game addiction and well-being among middle school children with structural heart disease and their typically developing peers?
3. What associations exist between well-being and computational thinking skills among middle school children with structural heart disease and their typically developing peers?

## 3. Method

### 3.1. Research Design

This study employed an exploratory comparative correlational design with group-based analyses. The primary aim was to examine associations within each group rather than to test between-group differences or interaction effects formally. No causal, mediational, or moderational hypotheses were tested. This design is appropriate for examining the strength and direction of naturally occurring associations among variables without manipulating study conditions [60]. In this context, the present study investigated the relationships among digital game addiction, computational thinking skills, and well-being in children with structural heart disease and their typically developing peers. Group-based analyses were included to descriptively examine whether the observed associations showed similar or differing patterns across the two groups. Because no experimental manipulation was conducted and the study focused on naturally occurring relationships rather than causal effects, an experimental design was not employed. Although an a priori power analysis was not conducted prior to data collection, a post hoc sensitivity analysis was applied to evaluate the minimum detectable effect sizes given the available sample. Based on the available sample sizes, a post hoc power analysis indicated that the study had approximately 80% power to detect correlations of r = 0.54 in the structural heart disease group (*n* = 25) and r = 0.49 in the comparison group (*n* = 30) at α = 0.05 (two-tailed). Therefore, smaller associations may not have been detected due to limited statistical power, and non-significant findings should be interpreted cautiously.

### 3.2. Sample

The study population consisted of middle school students aged 10–14 years with structural heart disease living in the city centre and districts of Afyonkarahisar, Türkiye. This developmental period is considered important for cognitive and social–emotional development and corresponds to an age range in which digital gaming habits become more pronounced [61,62].

The study sample was determined using purposive sampling. The inclusion criteria for the clinical group were children aged 10–14 years who were being followed by pediatric cardiology units at a university hospital during the 2024–2025 academic year and who had a diagnosis of structural heart disease (*n* = 25). The comparison group consisted of typically developing children in the same age range, with no chronic medical conditions, enrolled in public schools (*n* = 30). Purposive sampling was preferred because it enables the inclusion of participants with specific characteristics relevant to the study aims [63].

The modest sample size should be taken into account when interpreting the findings. Recruitment was limited by the relatively specific clinical population and the study’s inclusion criteria. Therefore, the findings may not be generalisable to broader populations and should be interpreted cautiously. Demographic characteristics of the children and their parents included in the study are presented in Table 1 and Table 2.

As shown in Table 1, among children with structural heart disease, 20.0% were female and 80.0% were male, whereas among those without structural heart disease, 56.7% were female and 43.3% were male. Regarding age distribution, the majority of children with structural heart disease were 14 years old (48.0%), followed by 10 years (20.0%), 12 years (16.0%), 11 years (8.0%), and 13 years (8.0%). In the group without structural heart disease, 30.0% of the children were 14 years old, 23.3% were 13 years old, 20.0% were 11 years old, 16.7% were 10 years old, and 10.0% were 12 years old. Although the groups were generally similar in age characteristics, some demographic differences, particularly in gender distribution, were observed.

In terms of birth order, 44.0% of the children with structural heart disease were firstborn, 24.0% were middleborn, and 32.0% were lastborn. Similarly, in the group without structural heart disease, 46.7% were firstborn, 30.0% were middleborn, and 23.3% were lastborn (Table 1). When parental characteristics were examined (Table 1), it was found that 52.0% of the mothers of children with structural heart disease were aged 40 years or older, 40.0% were between 30–39 years, and 8.0% were aged 29 years or younger. In the same group, 64.0% of the fathers were aged 40 years or older. In contrast, in the group without structural heart disease, the majority of both mothers and fathers were in the 30–39 age group.

Regarding educational level, the majority of mothers in both groups had primary education (with structural heart disease: 52.0%; without structural heart disease: 56.7%), followed by those with high school and university education. A similar pattern was observed for fathers’ educational levels, with primary and high school education being the most common in both groups (Table 1). An examination of maternal occupation indicated that the majority of mothers in both groups were homemakers (with structural heart disease: 72.0%; without structural heart disease: 80.0%). Regarding paternal occupation, the worker and self-employed categories were the most prominent in both groups (Table 1).

Clinical characteristics related to structural heart disease are presented in Table 2. Among the children with structural heart disease, 19 out of 25 had restrictions on sports and physical activities, whereas 11 children reported no such restrictions. Among those with restrictions, the majority had been restricted for four years or more.

### 3.3. Data Collection Instruments

In this study, data were collected using four instruments: (a) a Personal Information Form developed by the researchers to obtain general information about the children, (b) the Computational Thinking Skills Scale developed by Korkmaz, Çakır, and Özden [64] to assess children’s computational thinking skills, (c) the Digital Game Addiction Scale for Children developed by Hazar and Hazar [65] to evaluate digital game addiction, and (d) the EPOCH Well-Being Scale adapted into Turkish by Demirci and Ekşi [66] to assess children’s well-being.

### 3.4. Personal Information Form

The researchers developed the Personal Information Form to collect demographic and background information about the participating children. The form included items related to gender, birth order, parents’ age and educational level, parental occupation, history of cardiac surgery, time since diagnosis, and duration of restrictions on sports and physical activities.

### 3.5. Computational Thinking Skills Scale (CTSS)–Middle School Level

The Computational Thinking Skills Scale was developed by Korkmaz, Çakır, and Özden [64] to assess students’ levels of computational thinking. The scale consists of 21 items across five dimensions: creativity, algorithmic thinking, collaboration, critical thinking, and problem solving. Items are rated on a five-point Likert scale ranging from 1 (strongly disagree) to 5 (strongly agree), with higher total scores indicating higher levels of computational thinking skills. Confirmatory factor analysis results indicated that the scale’s factor structure provided an acceptable fit to the data. Cronbach’s alpha coefficients for the subscales ranged from 0.72 to 0.86. In contrast, the overall scale reliability was reported as 0.82. These findings indicate that the scale is a valid and reliable measurement tool for use at the middle school level.

### 3.6. Digital Game Addiction Scale for Children

The Digital Game Addiction Scale for Children was developed by Hazar and Hazar [65] to assess levels of digital game addiction among children. The scale was validated with a sample of 364 children aged 10–14 years. Exploratory factor analysis revealed a four-factor structure consisting of excessive gaming, loss of control, withdrawal, and neglect of social life. The scale includes 24 items rated on a five-point Likert scale (1 = strongly disagree, 5 = strongly agree). Confirmatory factor analysis indicated that the model demonstrated acceptable fit indices (KMO = 0.93; Bartlett’s test of sphericity, *p* < 0.001). Cronbach’s alpha coefficients ranged from 0.86 to 0.91 across the subscales. In addition, test–retest reliability analyses showed that the scale produced stable measurements over time.

### 3.7. Five-Dimensional Well-Being Model for Adolescents: EPOCH Scale

The EPOCH Well-Being Scale was originally developed by Kern et al. [67] and adapted into Turkish by Demirci and Ekşi [66]. The scale consists of 20 items and five dimensions: engagement, perseverance, optimism, connectedness, and happiness. Each item is rated on a five-point Likert scale ranging from 1 (strongly disagree) to 5 (strongly agree). Confirmatory factor analysis results indicated good model fit (χ^2^ = 381.29, *df* = 160, RMSEA = 0.074, CFI = 0.98). Cronbach’s alpha coefficients ranged between 0.72 and 0.88 for the subscales, while the overall internal consistency coefficient of the scale was reported as 0.95.

### 3.8. Data Collection Procedure

Prior to conducting the study, preliminary procedures were carried out to determine the study group. The voluntary participation of the children’s parents in the sample was considered essential. Therefore, parents who agreed to participate were first informed in detail about the study’s purpose, scope, procedures, and the measurement instruments to be used. Informed consent was obtained from the participating parents, and the researcher asked them to complete the measurement instruments. The data collection process was carried out in accordance with ethical principles, with due regard to confidentiality and participants’ rights.

Data were collected individually from the participating children in a quiet environment within the hospital or school setting, depending on group membership and participant availability. The questionnaires were completed by the children themselves under the researchers’ supervision. Prior to administration, participants were informed of the study’s purpose and instructed on how to complete the measures. Assistance was provided when clarification was needed, but no guidance related to item responses was given. The data collection process took approximately 20–25 min for each participant.

### 3.9. Data Analysis

The data obtained from the study were analysed using SPSS version 22. The internal consistency of the measurement instruments was examined using Cronbach’s alpha coefficients, and the normality of the data distribution was assessed with the Shapiro–Wilk test [68]. Frequency and percentage distributions were calculated for demographic characteristics and clinical variables. Because several variables did not meet normality assumptions, non-parametric analyses were preferred. Spearman’s rank-order correlation coefficient was used to examine the direction and strength of associations among the study variables [69]. In all statistical analyses, the level of statistical significance was set at *p* < 0.05 [70]. The Cronbach’s alpha reliability coefficients calculated for the sample are presented in Table 3.

The reliability coefficients were calculated as 0.878 for the Computational Thinking Skills Scale, 0.948 for the Digital Game Addiction Scale, and 0.907 for the Well-Being Scale. These results indicate that all three measurement instruments demonstrated high internal consistency within the sample [71]. In addition, effect size coefficients were calculated for group comparison analyses. Cohen’s *d* was used for variables that exhibited a normal distribution, whereas the *r* effect size (r = Z/√N) was calculated for variables that did not show a normal distribution. In interpreting effect sizes, the criteria proposed by Cohen [72] were applied: small ≈ 0.20, medium ≈ 0.50, and large ≈ 0.80 for Cohen’s *d*; and small ≈ 0.10, medium ≈ 0.30, and large ≈ 0.50 for *r* values [72].

Given the relatively small sample size and the number of statistical tests conducted, the results should be interpreted as exploratory. Therefore, non-significant findings should be interpreted cautiously, as limited statistical power may have reduced sensitivity to smaller relationships. Given the exploratory nature of the study and the relatively small sample size, multivariate analyses (e.g., regression models) were not conducted, as such models require larger samples to ensure stable and reliable estimates. Additionally, no correction procedures (e. g., Bonferroni adjustment) were applied, as the study aimed to explore potential associations rather than test a limited set of predefined hypotheses. However, this increases the risk of Type I error; therefore, the findings should be interpreted with caution.

## 4. Results

This study aimed to examine the relationships among digital game addiction, computational thinking skills, and well-being levels of middle school students with structural heart disease and their typically developing peers. In line with this aim, the study was structured around three sub-problems. The analyses related to the first sub-problem are presented in Table 4 and Table 5.

According to Table 4, among children with structural heart disease, excessive gaming was negatively associated with creativity (r = −0.552), and overall computational thinking skills (r = −0.463) (*p* < 0.05). Similarly, neglect of social life showed a negative association with collaboration (r = −0.410) (*p* < 0.05). No other statistically significant associations were observed between digital game addiction and computational thinking sub-dimensions.

In addition, the effect size for the observed difference in total computational thinking scores was Cohen’s d = 0.61, which corresponds to a moderate effect size according to Cohen’s [72] classification. When the sub-dimensions were examined, moderate effect sizes were found for creativity (r = 0.31) and collaboration (r = 0.36); small effect sizes were observed for algorithmic thinking (r = 0.22) and critical thinking (r = 0.24); and a very small effect size was identified for problem solving (r = 0.14). These results suggest that the differences observed in certain sub-dimensions are not only statistically significant but also have practical relevance, given their moderate effect sizes. Similarly, effect sizes for the sub-dimensions of digital game addiction ranged from small to moderate. Effect sizes approaching the moderate level were found for *loss of control* (r = 0.33), excessive gaming (r = 0.28), and total digital game addiction score (r = 0.28). In contrast, a small effect size was observed for the withdrawal sub-dimension (r = 0.12). These findings indicate that although some sub-dimensions exhibit effects beyond statistical significance, the overall magnitude of the effect remains limited.

According to Table 5, it was found that the critical thinking sub-dimension of children without structural heart disease was negatively and significantly associated with the loss of control, withdrawal, and neglect of social life sub-dimensions of digital game addiction, as well as with overall digital game addiction (r = −0.451) (*p* < 0.05). In addition, significant negative relationships were identified between the withdrawal sub-dimension and creativity (r = −0.369), collaboration (r = −0.462), and overall computational thinking skills (r = −0.515) (*p* < 0.05). Furthermore, statistically significant negative relationships were observed between loss of control and algorithmic thinking (r = −0.377), and between neglect of social life and collaboration (r = −0.401) (*p* < 0.05). These findings indicate that, among typically developing children, increases in digital game addiction scores are associated with negative effects on several sub-dimensions of computational thinking skills, particularly critical thinking and collaboration. However, no statistically significant relationship was found between the problem-solving sub-dimension and digital game addiction (*p* > 0.05). The analyses related to the second sub-problem are presented in Table 6 and Table 7.

According to Table 6, no statistically significant relationships were found between any sub-dimensions of digital game addiction and the sub-dimensions of connectedness, engagement, happiness, optimism, perseverance, or the overall well-being level of children with structural heart disease (*p* > 0.05). An examination of the correlation coefficients revealed that they were at very low levels, indicating that the variables operated largely independently of one another. These findings suggest that, in children with structural heart disease, tendencies toward digital game addiction do not have a determining effect on children’s overall perceptions of well-being. In addition, the effect size for the total well-being score was d = 0.19, indicating a small effect. Effect sizes for the sub-dimensions were also generally small (r ≅ 0.08–0.24). Although statistically significant differences were observed between groups in some analyses, these results indicate that the magnitude of the differences remains limited and that the practical impact of these differences may be relatively low.

According to Table 7, no statistically significant relationships were found between digital game addiction and any of the well-being sub-dimensions or the total well-being score among children without structural heart disease (*p* > 0.05). The findings indicate that digital gaming habits of typically developing children are not significantly associated with their well-being components. The analyses related to the third sub-problem are presented in Table 8 and Table 9.

According to Table 8, statistically significant, positive, and moderate correlations were found between the connectedness sub-dimension of children with structural heart disease and algorithmic thinking (r = 0.554), critical thinking (r = 0.526), and overall computational thinking skills (r = 0.456) (*p* < 0.05). In addition, a statistically significant positive relationship was observed between perseverance and critical thinking (r = 0.448) (*p* < 0.05). These findings indicate that as children with structural heart disease develop stronger connections with their social environment (connectedness), their ability to manage logical processes and engage in critical analysis also increases. However, no statistically significant relationships were found between computational thinking skills and the dimensions of engagement, happiness, and optimism (*p* > 0.05).

According to Table 9, when the relationship between computational thinking skills and well-being levels of children without structural heart disease was examined, the perseverance sub-dimension was found to be positively and statistically significantly associated with creativity (r = 0.394), collaboration (r = 0.422), critical thinking (r = 0.569), and overall computational thinking skills (r = 0.412) (*p* < 0.05). In addition, significant positive relationships were identified between overall well-being and creativity (r = 0.370), collaboration (r = 0.389), and critical thinking (r = 0.407) (*p* < 0.05). These findings indicate that as psychological resilience and perseverance levels increase among children without structural heart disease, their computational thinking skills also tend to improve. However, no statistically significant relationships were observed for the algorithmic thinking and problem-solving sub-dimensions (*p* > 0.05).

## 5. Discussion

In this study, the relationships among digital game addiction, computational thinking skills, and well-being levels of middle school students with structural heart disease and their typically developing peers were examined within the framework of three sub-problems. The findings revealed more pronounced relationships between digital game addiction and cognitive skills, whereas the associations with well-being variables were limited and relatively weak. Specifically, while significant associations were identified between digital game addiction and computational thinking skills, the relationships involving well-being components remained limited. The observed effect sizes were generally small to moderate, indicating that the associations identified in this study are limited in magnitude and should not be interpreted as strong relationships. These results suggest that digital gaming behaviours are more strongly associated with socio-cognitive components such as creativity and collaboration than with general well-being indicators.

When the findings related to the first sub-problem of the study were examined, it was revealed that as the digital game addiction sub-dimensions of excessive gaming and neglect of social life increased, computational thinking skills—particularly creativity, collaboration, and overall computational thinking—decreased. Especially among children with structural heart disease, moderate and negative relationships were identified between excessive gaming and both creativity and total computational thinking scores. Similarly, a negative association was found between neglect of social life and collaboration skills. These findings suggest that increased gaming duration may be associated with lower levels of productive thinking, collaborative work, and the generation of alternative solutions to problem situations. Previous research supports this interpretation, indicating that excessive and uncontrolled digital game use may have adverse effects on children’s attention, academic performance, and social adjustment skills [24,27,29,73,74]. One possible explanation for this pattern is that children in the disease group experience limitations in physical activity and spend a substantial amount of time in the home environment, which may be associated with greater engagement with digital games. However, when such use is unstructured and poorly regulated, it may shift from a potentially engaging activity into more passive consumption rather than supporting cognitive development. Studies have also reported that insufficient parental monitoring and low levels of digital parenting awareness increase the risk of addictive behaviours and developmental vulnerabilities in children [30,65]. Recent international research on parental involvement in digital gaming suggests that its effects are not uniform and may vary depending on the type of mediation strategy. For instance, restrictive mediation may reduce excessive gaming, whereas active mediation and co-use strategies may support healthier engagement with digital games and preserve their potential developmental benefits.

Furthermore, adolescents’ motivations for gaming, particularly escapism as a distinct motivational profile, have been shown to play a critical role in the development of problematic gaming behaviours [3]. Repetitive game experiences based on predefined, ready-made scenarios may reduce opportunities for creative production and analytical reasoning, which may be associated with lower levels of computational thinking skills [75,76]. Moreover, the observed pattern is consistent with previous findings linking problematic gaming behaviour with attention and academic difficulties [31,77]. Finally, the effect size findings indicated that the associations for algorithmic thinking and problem-solving sub-dimensions remained small, suggesting that digital game use is not equally related to all cognitive domains. These findings highlight the importance of considering parental mediation not only as a control mechanism but also as a multidimensional construct that may influence different gaming motivations in distinct ways.

In contrast, among children without structural heart disease, the observed associations were distributed across a broader range of cognitive skills. For instance, the negative associations between digital game addiction scores and critical thinking, collaboration, and overall computational thinking indicate that excessive engagement with digital games may be related to lower levels of cognitive performance not only in children with structural heart disease but also in typically developing children. The findings of the present study are consistent with previous research demonstrating that higher levels of problematic gaming are associated with attention problems, academic difficulties, and social withdrawal behaviours [31,77]. In particular, increases in addiction-related symptoms such as loss of control and withdrawal may be related to impairments in higher-order cognitive skills, including attention regulation, planning, and logical decision-making. This pattern suggests that when digital games are used primarily for escape or habitual engagement, cognitive functioning may be negatively associated with them. Notably, there is no significant association between digital game addiction and the problem-solving sub-dimension. Previous studies have similarly reported that digital game addiction does not show consistent associations across all cognitive domains [23]. When both the existing literature and the present study’s findings are considered, this result may be explained by the fact that problem-solving is a more general skill shaped by daily life experiences and school-based activities.

When the findings related to the second sub-problem of the study were examined, it was found that there was no statistically significant association between digital game addiction and well-being levels in either children with structural heart disease or those without structural heart disease. In other words, contrary to expectations, no statistically significant associations were found between digital game addiction and well-being in either group. This finding suggests that, in this sample, no linear relationship was detected between these variables. However, this result should not be interpreted as evidence of the absence of a relationship. Rather, it may reflect the multidimensional and complex structure of well-being. Several factors may account for this finding. First, well-being is a multidimensional construct, and different dimensions may not be equally sensitive to digital game-related behaviours. Second, the EPOCH scale, while widely used, may not capture more context-specific aspects of well-being related to digital media use. Third, developmental factors in middle school children may influence how digital game use relates to subjective well-being. Finally, the relatively small sample size limited the statistical power to detect existing associations. In particular, for children with structural heart disease, well-being levels are influenced by a wide range of factors, including health status, family support, treatment processes, social acceptance, and overall life experiences. Previous studies conducted with children diagnosed with structural heart disease similarly indicate that quality of life and psychosocial well-being are more strongly associated with medical and family-related variables than with behavioural factors such as digital game use [14,16]. Therefore, for children coping with a chronic health condition, psychological well-being may be shaped primarily by health-related and family-based support mechanisms rather than by digital gaming behaviours. The absence of statistically significant associations between digital game addiction and well-being is theoretically meaningful and warrants careful interpretation. One possible explanation lies in the multidimensional structure of well-being, which is influenced by a wide range of factors beyond digital behaviours, including family support, health status, and social relationships. In this context, digital gaming alone may not constitute a sufficiently strong or direct determinant of overall well-being.

Furthermore, moderate levels of digital game use may serve adaptive functions such as stress relief, social interaction, or emotional regulation, thereby weakening the expected negative association with well-being. This interpretation aligns with previous literature suggesting that the impact of digital gaming on psychological outcomes depends on the intensity, purpose, and context of use rather than mere exposure. Additionally, any psychosocial implications regarding the impact of digital game addiction on well-being should be interpreted with caution, as present findings do not provide direct empirical support for such claims.

Similarly, the absence of significant associations in children without structural heart disease suggests that digital games are not necessarily related to negative psychological outcomes in all cases. Children may use digital games for entertainment, socialisation, or stress reduction. Therefore, unless gaming behaviour exceeds a certain threshold, it may not constitute a substantial risk factor for well-being. In this respect, establishing a direct and linear relationship between symptoms of digital game addiction and subjective well-being is not always possible [24]. Moreover, the limited number of significant associations and the generally small effect sizes observed in well-being variables indicate that digital game addiction is not a strong determinant of psychological well-being in this study. This is particularly evident in children with structural heart disease, for whom well-being is more closely associated with factors such as health status and family support [14]. Accordingly, when the multidimensional nature of well-being is taken into account, digital gaming behaviours may be related to only a limited aspect of this broader construct.

When the findings related to the third sub-problem of the study were examined, positive associations were observed between computational thinking skills and well-being levels, particularly in the social and psychological dimensions. The literature similarly emphasises that cognitive skills, social adjustment, and psychological resilience are interrelated constructs that support one another and tend to develop concurrently [44,46]. In children with structural heart disease, the associations between connectedness and perseverance, and between algorithmic thinking, critical thinking, and overall computational thinking, suggest that cognitive skills develop in conjunction with social adjustment and psychological resilience. Children who possess logical reasoning, planning, and systematic thinking skills may also show higher levels of coping and social engagement. Supporting this interpretation, previous research has shown that computational thinking is related to problem solving, collaboration, and higher-order thinking skills, which are associated with both academic and social development [35]. From this perspective, particularly for children facing health-related challenges, cognitive competence may be associated with psychological empowerment. In brief, feelings of “being able to succeed” and “having control” may be related to both social connectedness and internal resilience.

In children without structural heart disease, the finding that well-being—particularly the perseverance dimension—is associated with creativity, collaboration, and critical thinking is noteworthy. This pattern suggests that cognitive flexibility and productive thinking may co-occur with psychological resilience. It is expected that children who can express themselves effectively, collaborate within groups, and engage in critical thinking may report higher life satisfaction and perseverance in pursuing their goals. From this perspective, computational thinking skills can be considered not only as academic competencies but also as variables that support psychosocial development. The absence of significant relationships in the problem-solving dimension may be related to the more general and multifaceted nature of this skill compared to other sub-dimensions. Indeed, some studies have reported that consistent relationships are not observed across all cognitive and psychosocial dimensions, and that problem-solving represents a broader construct shaped by multiple environmental experiences [23]. Given the exploratory and correlational nature of the analyses and the absence of correction for multiple testing, these findings should be considered preliminary. Therefore, the non-significant findings should not be interpreted as evidence of the absence of a relationship, but rather as an indication that no detectable associations were observed in the present sample.

The present study provides preliminary empirical evidence regarding the associations among digital game addiction, computational thinking skills, and well-being in middle school children with and without structural heart disease. However, given the cross-sectional and correlational design, the findings should not be interpreted as evidence of causal or explanatory relationships. Rather, the results should be considered as exploratory, highlighting potential relationships that warrant further investigation. The findings should be interpreted cautiously, particularly given the limited statistical power of the sample. Therefore, the results should be considered preliminary and warrant replication with larger samples. Future research should employ a longitudinal or experimental design to examine the directionality and potential causal mechanisms underlying these associations.

The findings of the present study do not provide evidence for causal, mediating, or moderating relationships. Therefore, interpretations of the potential protective or buffering roles of computational thinking should be considered speculative and require further investigation.

## 6. Conclusions

In this study, the relationships among digital game addiction, computational thinking skills, and well-being levels of middle school students with structural heart disease and their typically developing peers were examined comparatively. The findings indicated that as digital game addiction increased, declines were observed, particularly in certain dimensions of computational thinking skills. In contrast, no direct or strong relationship was found between digital game addiction and overall well-being levels. An examination of effect sizes revealed moderate effects for the total computational thinking score, small-to-moderate effects for the dimensions of digital game addiction, and small effects for the well-being variables. These results suggest that the observed relationships differ not only in statistical significance but also in magnitude across variables. In addition, computational thinking skills were found to be positively associated with well-being components, including social connectedness, perseverance, and psychological resilience. Accordingly, the findings demonstrate that digital games do not function merely as tools for entertainment in children’s lives but constitute an experiential domain closely related to both cognitive and psychosocial development. However, uncontrolled and excessive gaming behaviours may limit higher-order cognitive skills such as creativity, critical thinking, and collaboration.

When evaluated specifically in the context of children with structural heart disease, the findings suggest that increased engagement with digital environments due to physical limitations may pose risks for cognitive and socio-emotional development when digital game use becomes passive and addiction-oriented. Nevertheless, the fact that effect sizes generally remained within the small-to-moderate range suggests that the relationship between digital game use and cognitive and psychosocial development is relatively small in magnitude. Moreover, the reporting of effect sizes in this study enabled the findings to be interpreted not only in terms of statistical significance but also in terms of their practical relevance. The results revealed moderate effects, particularly for cognitive variables, whereas psychosocial variables demonstrated small effect sizes, suggesting that the impact of digital gaming behaviours may vary across developmental domains.

In conclusion, this study provides preliminary evidence of associations among digital game addiction, computational thinking skills, and well-being in middle school children with and without structural heart disease. The findings indicate that digital game addiction is generally associated with lower computational thinking skills, while computational thinking shows weak to moderate positive associations with certain dimensions of well-being. However, no consistent relationship was observed between digital game addiction and well-being. Given the exploratory correlational design, small sample size, and multiple testing considerations, these findings should be interpreted cautiously and should not be considered causal. Rather than providing definite conclusions, the results offer initial insights into the complex relationships among digital behaviours, cognitive skills, and well-being in childhood. Future studies with larger, more representative samples are needed to clarify these relationships further and test more complex explanatory models.

## 7. Recommendations

Based on the study’s findings, several cautious recommendations can be made. First, the observed negative associations between digital game addiction and computational thinking skills suggest that educational practices should encourage balanced digital engagement and structured learning activities that support problem-solving and creative thinking. Second, the generally weak associations between digital game addiction and well-being suggest that multiple contextual factors may influence children’s well-being; therefore, interventions should adopt a broader psychosocial perspective rather than focusing solely on gaming behaviour. Third, the positive associations between computational thinking and certain well-being dimensions suggest that integrating computational thinking activities into educational settings may support adaptive cognitive and emotional development.

However, given the exploratory and correlational nature of the study, these recommendations should be interpreted cautiously and considered as preliminary suggestions for future research and educational practice.

## 8. Limitations

Despite its contributions, this study has several limitations that should be considered when interpreting the findings. First, the sample size was relatively small, which may have limited statistical power and reduced the likelihood of detecting smaller associations. The study was conducted in Afyonkarahisar, where a single specialist physician provides pediatric cardiology services. Accordingly, access to children with structural heart disease was limited to a single clinical setting, and data collection was conducted over 7 months. Within this timeframe, the number of eligible participants who could be reached remained limited.

Second, participation in the study was entirely voluntary. Given the sensitive nature of chronic health conditions in children, some families chose not to participate, further limiting the sample size. Therefore, the sample may not be fully representative of broader populations of children with or without structural heart disease, and the findings should be interpreted cautiously.

Third, the two comparison groups were not fully balanced in their demographic composition, particularly in gender distribution. This imbalance may have influenced some of the observed associations. Previous research has shown that gender differences may influence both digital gaming behaviours and psychosocial outcomes during middle childhood and early adolescence. In general, boys tend to report higher levels of digital gaming engagement and problematic gaming behaviours, whereas girls often report comparatively higher levels of social connectedness and emotional well-being [31,77]. Therefore, the predominance of boys in the structural heart disease group and the relatively higher proportion of girls in the comparison group may have partially shaped some of the observed association patterns. For example, the stronger negative associations observed between digital game addiction and computational thinking dimensions in the structural heart disease group may partly reflect gender-related differences in gaming intensity and gaming motivations. Similarly, differences in well-being-related associations may also have been influenced by gender-related psychosocial characteristics reported in previous literature. Accordingly, the findings should be interpreted cautiously, as some observed group differences may reflect demographic composition in addition to disease-related factors.

Fourth, potentially relevant control variables such as socioeconomic status, parental mediation of technology use, and total screen time were not included in the analyses. These unmeasured variables may have contributed to the observed relationships.

Another limitation concerns the research design’s cross-sectional and correlational nature. As the study aimed to examine relationships among variables at a single point in time, causal inferences about the direction of these relationships cannot be made. Longitudinal or experimental designs may provide a more comprehensive understanding of how digital game addiction, computational thinking skills, and well-being interact over time.

Another limitation is reliance on multiple bivariate correlation analyses. Conducting a large number of statistical tests may increase the risk of Type I error (false-positive findings). As no correction procedures were applied, some statistically significant results should be interpreted cautiously. Also, there is no a priori power analysis. Given the relatively small sample size, the study may be underpowered to detect small-to-moderate associations. Therefore, the precision of the estimated correlations may be limited, and some non-significant findings may reflect insufficient statistical power rather than the true absence of relationships. Consequently, the reported coefficients should be interpreted as preliminary estimates, with potential uncertainty regarding the stability of the effect size.

Finally, data were collected through self-report scales, which may be subject to response bias and social desirability effects. This methodological approach may introduce common-method bias and social desirability effects, as participants may overreport socially desirable behaviours and underreport undesirable ones. As a result, the observed relationships among digital game addiction, computational thinking skills, and well-being may partially reflect participants’ subjective perceptions rather than fully objective behavioural patterns. This limitation should be considered when interpreting the strength and direction of the associations reported in the present study. Although the measurement instruments used in the study demonstrated strong reliability and validity, future research may benefit from incorporating multi-informant data sources (e.g., parents, teachers, or clinical observations) and mixed-method approaches to obtain a more comprehensive perspective. Future studies would benefit from incorporating multi-informant data sources such as teacher reports, parental evaluations, or objective digital usage logs to reduce potential bias.

## Figures and Tables

**Table 1:** Demographic characteristics of the study group.

		Children with Structural Heart Disease			
		Yes		No	
		N	%	N	%
Gender	Female	5	20.0%	17	56.7%
	Male	20	80.0%	13	43.3%
Age (years)	10	5	20.0%	5	16.7%
	11	2	8.0%	6	20.0%
	12	4	16.0%	3	10.0%
	13	2	8.0%	7	23.3%
	14	12	48.0%	9	30.0%
Birth Order	Firstborn	11	44.0%	14	46.7%
	Middle born	6	24.0%	9	30.0%
	Last born	8	32.0%	7	23.3%
Mother’s Age	29 and below	2	8.0%	2	6.7%
	30–39	10	40.0%	18	60.0%
	40 and above	13	52.0%	10	33.3%
Father’s Age	29 and below	1	4.0%	2	6.7%
	30–39	8	32.0%	12	40.0%
	40 and above	16	64.0%	16	53.3%
Mother’s Education Level	Primary	13	52.0%	17	56.7%
	High School	8	32.0%	8	26.7%
	University	4	16.0%	5	16.7%
	Postgraduate Education	0	0.0%	0	0.0%
Father’s Education Level	Primary	9	36.0%	11	36.7%
	High School	8	32.0%	8	26.7%
	University	7	28.0%	10	33.3%
	Postgraduate Education	1	4.0%	1	3.3%
Mother’s Occupation	Homemaker	18	72.0%	24	80.0%
	Civil servant	2	8.0%	3	10.0%
	Worker	4	16.0%	1	3.3%
	Self-employed	1	4.0%	2	6.7%
Father’s Occupation	Retired	5	20.0%	1	3.3%
	Civil servant	5	20.0%	7	23.3%
	Worker	7	28.0%	13	43.3%
	Self-employed	8	32.0%	9	30.0%

**Table 2:** Clinical characteristics of children with structural heart disease.

		Children with Structural Heart Disease	
		Yes	
		N	%
Time since diagnosis	1 year ago	2	8.0%
	2 years ago	3	12.0%
	3 years ago	3	12.0%
	4 years or more ago	17	68.0%
	No restriction	11	36.7%
Restricted from sports and similar physical activities	For 1 year	3	10.0%
	For 2 years	4	13.3%
	For 3 years	2	6.7%
	For 4 years or more	10	33.3%
History of cardiac surgery	Yes	8	32.0%
	No	17	68.0%

**Table 3:** Cronbach’s alpha reliability coefficients.

Scales	Cronbach’s Alpha	Number of Items
Computational Thinking Skills Scale	0.878	21
Digital Game Addiction Scale	0.948	24
Well-Being Scale	0.907	20

**Table 4:** Spearman correlations between DGA and CT skills among children with structural heart disease.

Structural Heart Disease: Yes		Neglect of Social Life	Excessive Gaming	Loss of Control	Withdrawal	Digital Game Addiction
Creativity	r	−0.373	−0.552 *	−0.214	−0.264	−0.327
	*p*	0.066	0.004	0.305	0.202	0.111
Algorithmic thinking	r	−0.243	−0.280	−0.033	−0.242	−0.212
	*p*	0.242	0.176	0.876	0.245	0.310
Collaboration	r	−0.410 *	−0.295	−0.248	−0.218	−0.267
	*p*	0.042	0.152	0.232	0.294	0.197
Critical Thinking	r	−0.122	−0.375	−0.188	−0.076	−0.146
	*p*	0.561	0.065	0.368	0.719	0.487
Problem Solving	r	0.018	−0.129	−0.077	0.010	0.008
	*p*	0.930	0.538	0.716	0.963	0.971
Computational Thinking Skills	r	−0.330	−0.463 *	−0.217	−0.222	−0.270
	*p*	0.107	0.020	0.298	0.286	0.191

* indicates statistically significant correlations at the 0.05 level (*p* < 0.05)

**Table 5:** Spearman correlation analysis of the relationship between DGA and CT skills in children without structural heart disease.

Structural Heart Disease: No		Neglect of Social Life	Excessive Gaming	Loss of Control	Withdrawal	Digital Game Addiction
Creativity	r	−0.327	−0.169	−0.298	−0.369 *	−0.261
	*p*	0.078	0.371	0.110	0.045	0.163
Algorithmic thinking	r	−0.193	0.043	−0.377 *	−0.284	−0.133
	*p*	0.307	0.820	0.040	0.128	0.483
Collaboration	r	−0.401 *	−0.250	−0.318	−0.462 *	−0.345
	*p*	0.028	0.184	0.086	0.010	0.062
Critical Thinking	r	−0.452 *	−0.352	−0.491 *	−0.452 *	−0.451 *
	*p*	0.012	0.056	0.006	0.012	0.012
Problem Solving	r	0.201	0.227	0.180	−0.023	0.174
	*p*	0.288	0.227	0.340	0.904	0.358
Computational Thinking Skills	r	−0.239	−0.146	−0.320	−0.515 *	−0.269
	*p*	0.204	0.440	0.084	0.004	0.150

* indicates statistically significant correlations at the 0.05 level (*p* < 0.05)

**Table 6:** Spearman correlation analysis of the relationship between DGA and well-being scores of children with structural heart disease.

Structural Heart Disease: Yes		Neglect of Social Life	Excessive Gaming	Loss of Control	Withdrawal	Digital Game Addiction
Connectedness	r	0.054	−0.031	0.126	0.223	0.148
	*p*	0.799	0.881	0.548	0.285	0.481
Engagement	r	−0.027	−0.111	−0.023	0.133	−0.017
	*p*	0.898	0.598	0.914	0.527	0.935
Happiness	r	0.077	−0.007	0.069	0.240	0.119
	*p*	0.715	0.973	0.741	0.247	0.570
Optimism	r	−0.115	−0.099	0.016	0.120	−0.029
	*p*	0.583	0.639	0.938	0.567	0.891
Perseverance	r	−0.188	−0.365	−0.231	−0.078	−0.220
	*p*	0.369	0.073	0.266	0.710	0.291
Well-being level	r	−0.092	−0.166	−0.055	0.113	−0.035
	*p*	0.661	0.429	0.795	0.592	0.867

**Table 7:** Spearman correlation analysis of the relationship between DGA and well-being scores of children without structural heart disease.

Structural Heart Disease: No		Neglect of Social Life	Excessive Gaming	Loss of Control	Withdrawal	Digital Game Addiction
Connectedness	r	−0.090	0.003	0.085	−0.040	0.040
	*p*	0.636	0.986	0.653	0.833	0.833
Engagement	r	−0.151	0.005	−0.022	0.088	0.002
	*p*	0.427	0.981	0.907	0.644	0.991
Happiness	r	−0.025	−0.022	0.027	0.016	0.053
	*p*	0.894	0.908	0.887	0.931	0.782
Optimism	r	−0.101	−0.209	−0.190	−0.045	−0.133
	*p*	0.597	0.267	0.316	0.815	0.482
Perseverance	r	−0.205	−0.217	−0.180	−0.205	−0.232
	*p*	0.276	0.250	0.340	0.277	0.217
Well-being level	r	−0.119	−0.082	−0.081	−0.023	−0.046
	*p*	0.531	0.668	0.669	0.902	0.808

**Table 8:** Spearman correlation analysis of the relationship between CT skills and well-being scores of children with structural heart disease.

Structural Heart Disease: Yes		Connectedness	Engagement	Happiness	Optimism	Perseverance	Well-Being Level
Creativity	r	0.378	0.262	0.122	0.241	0.321	0.322
	*p*	0.062	0.206	0.560	0.245	0.118	0.117
Algorithmic thinking	r	0.554 *	0.062	0.157	0.121	0.282	0.252
	*p*	0.004	0.767	0.453	0.566	0.172	0.224
Collaboration	r	0.365	0.059	0.307	0.159	0.351	0.315
	*p*	0.073	0.780	0.136	0.448	0.086	0.125
Critical Thinking	r	0.526 *	0.090	0.116	−0.033	0.448 *	0.267
	*p*	0.007	0.670	0.581	0.875	0.025	0.196
Problem Solving	r	−0.344	−0.156	−0.326	−0.386	−0.210	−0.330
	*p*	0.092	0.456	0.111	0.057	0.313	0.107
Computational Thinking Skills	r	0.456 *	0.045	0.082	0.021	0.354	0.224
	*p*	0.022	0.830	0.698	0.919	0.082	0.281

* indicates statistically significant correlations at the 0.05 level (*p* < 0.05).

**Table 9:** Spearman correlation analysis of the relationship between CT and well-being scores of children without structural heart disease.

Structural Heart Disease: No		Connectedness	Engagement	Happiness	Optimism	Perseverance	Well-Being Level
Creativity	r	0.246	0.267	0.348	0.247	0.394 *	0.370 *
	*p*	0.189	0.153	0.059	0.189	0.031	0.044
Algorithmic thinking	r	0.121	0.122	0.163	0.212	0.350	0.263
	*p*	0.523	0.521	0.389	0.260	0.058	0.160
Collaboration	r	0.275	0.233	0.314	0.246	0.422 *	0.389 *
	*p*	0.142	0.216	0.091	0.189	0.020	0.034
Critical Thinking	r	0.332	0.298	0.246	0.354	0.569 *	0.407 *
	*p*	0.073	0.110	0.190	0.055	0.001	0.026
Problem Solving	r	−0.258	0.034	−0.023	−0.020	−0.193	−0.085
	*p*	0.168	0.860	0.904	0.915	0.308	0.654
Computational Thinking Skills	r	0.133	0.212	0.223	0.227	0.412 *	0.309
	*p*	0.484	0.261	0.236	0.228	0.024	0.097

* indicates statistically significant correlations at the 0.05 level (*p* < 0.05).

## Data Availability

The data presented in this study are available from the corresponding author upon reasonable request. The data are not publicly available due to ethical restrictions and participant confidentiality.
